# Blood brain barrier disruption in cerebral malaria: Beyond endothelial cell activation

**DOI:** 10.1371/journal.ppat.1007786

**Published:** 2019-06-27

**Authors:** Arnulfo Tunon-Ortiz, Tracey J. Lamb

**Affiliations:** 1 Department of Pathology, University of Utah, Salt Lake City, Utah, United States of America; 2 Neurosciences Graduate Program, University of Utah, Salt Lake City, Utah, United States of America; University of Wisconsin Medical School, UNITED STATES

## Blood brain barrier breakdown in cerebral malaria: Beyond endothelial cell activation

Breakdown of the blood brain barrier (BBB) is a feature of cerebral malaria (CM), a manifestation of infection with *Plasmodium falciparum* parasites that currently has a 20% fatality rate and disproportionately affects children under 5 years old. Despite *Plasmodium* parasites being blood restricted, the BBB becomes disrupted when the brain microvascular endothelial cells (BMECs) lining the microvasculature of the brain are activated in response to sequestration of *Plasmodium*-infected red blood cells (iRBCs), as shown by in vitro co-culture assays of BMECs and iRBCs[1]. As part of the neurovascular unit (NVU), BMEC junctions are normally controlled by pericytes and astrocytes. Thus, barrier breakdown in malaria involves tripartite failure of these cellular players to maintain an intact barrier.

## What is the NVU?

**Fig 1:**
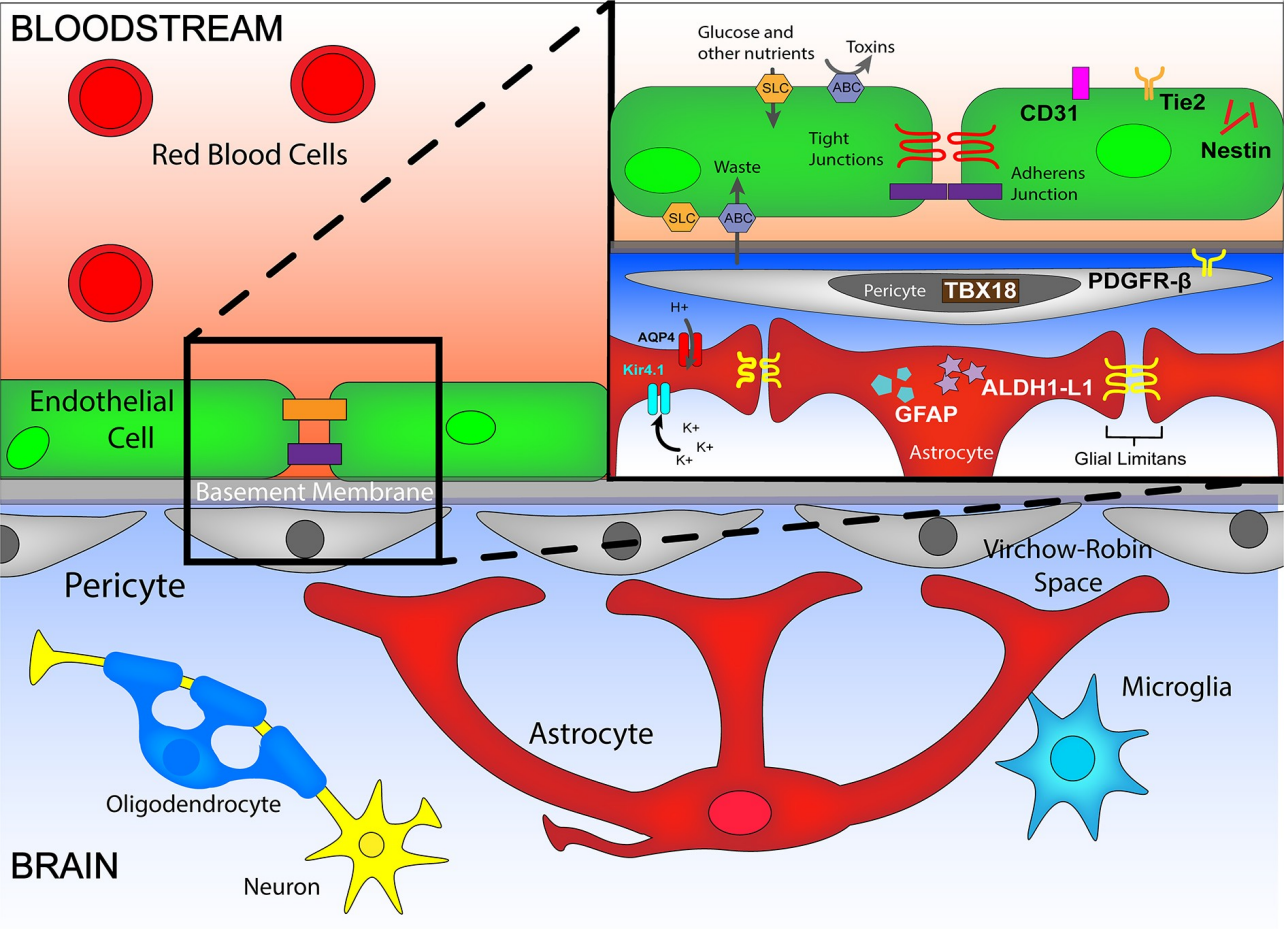
The NVU and its major components. The NVU is composed of endothelial cells, basement membrane, astrocytes, pericytes, and the distal neurons, microglia, and oligodendrocytes. Endothelial cells form the BBB via tight and adherens junctions. The SLC superfamily import proteins allow nutrients to cross the BBB, while the ABC superfamily of export proteins prevent toxins and expel waste in endothelial cells. Astrocytes contribute to regulating traffic at the BBB through various methods; among them is modulation of bulk flow via AQP4 and Kir4.1. Key cellular markers that identify the major cell types that make up the NVU are Tie 2, PECAM-1/CD31 and Nestin (endothelial cells), PDGRB and Tbx-18 (pericytes), and GFAP and ALDH1-L1 (astrocytes). ABC, ATP-binding cassette transporter; ALDH1-L1, aldehyde dehydrogenase 1 family member L1; AQP4, aquaporin-4; BBB, blood brain barrier; CD31, cluster of differentiation 31; GFAP, glial fibrillary acidic protein; Kir4.1, potassium inward rectifier channel 4.1; NVU, neurovascular unit; PDGRB, platelet-derived growth factor receptor-β; PECAM, platelet endothelial adhesion molecule; SCL, solute carrier; Tbx, T-box transcription factor; Tie 2, Angiopoietin-1 receptor/Tyrosine-protein kinase receptor TIE-2.

The BBB keeps the central nervous system (CNS) separated from the circulatory system. This selective semipermeable barrier is formed by specialized BMECs that regulate bidirectional traffic of molecules into and out of the CNS via tight and adherens junctions. The endothelium is further enveloped by pericytes and astrocyte endfeet that are regulators of the the formation and maintenance of the barrier’s integrity [2, 3]. These cells, along with more distal neurons and microglia that are separated from the barrier by the perivascular Virchow-Robin space, form a dynamic and functional unit termed the NVU (**Fig 1**) [4]. The NVU is a primary point of interaction for the peripheral immune system and the CNS.

## How do pericytes and astrocytes regulate BBB permeability under steady-state conditions?

**Fig 2:**
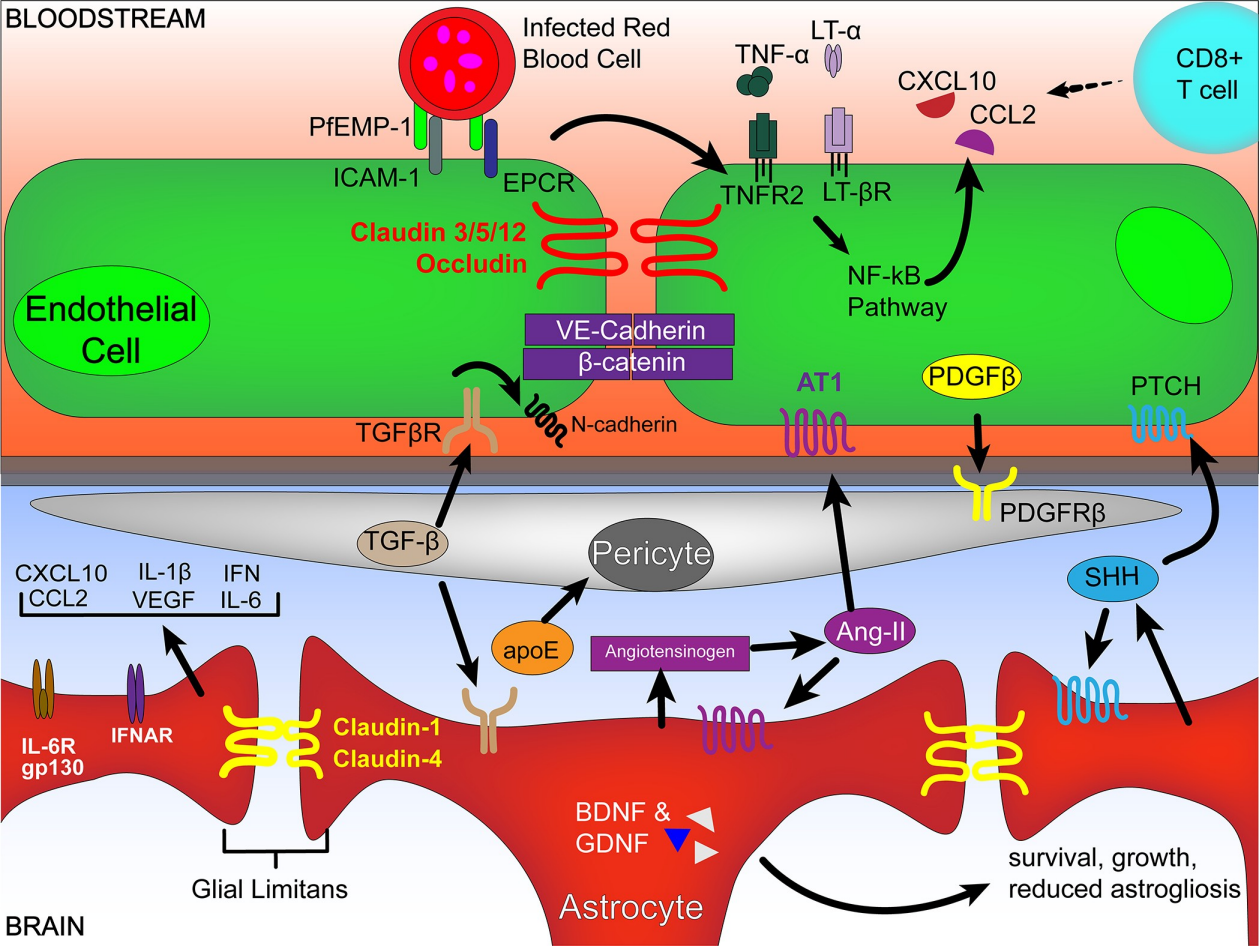
Modulators of endothelial cell cross talk with pericytes and astrocytes that may be relevant in BBB disruption during CM. Surface adhesion molecule ICAM-1 and EPCR are known molecules facilitating binding of *Pf*EMP-1 expressed on iRBCs. Induction of a signaling cascade triggers the transcription of TNF family members and chemokines. Binding of TNFR-2 and LT-βR activates the NF-_κ_B pathway, inducing the CXCL10 and CCL2 recruit immune cells, such as CD8^+^ T cells. Barrier integrity is maintained by tight junction proteins (claudins and occludins) and adherens junction proteins (cadherins and catenins). Pericytes are recruited and maintained at the BBB by the PDGFβ-PDGFRβ signaling axis. Pericyte production of TGF-β promotes adhesion of endothelial cells to the basement membrane via induction of N-cadherin and up-regulates claudin-5 as well as down-regulates the inflammatory capacity of astrocytes. apoE is hypothesized to down-regulate pericyte-induced regulation of endothelial cell tight junctions. Astrocytes can be pro-inflammatory, secreting a variety of cytokine and chemokines that include CXCL10, CCL2, IL-6, IL-1β, IFNs, and growth factors such as VEGF. The expression of cytokine receptors such as the IL-6R/gp130 complex and IFNAR facilitate responses of astrocytes in the context of an inflammatory response. During inflammation, astrocytes form a physical barrier at the BBB via claudin 1/4 in a structure called the glial limitans perivascularis, which regulates immune cell and molecule trafficking. Astrocytes are implicated in regulating BBB integrity via Ang-I/II derived from secreted angiotensinogen. Ang-II promotes the formation and maintenance of endothelial tight junctions via binding to the AT-1 expressed on endothelial cells. SHH is also secreted from astrocytes and has been shown to up-regulate occludin and claudin-5 on BMECs by signaling through the PTCH1 receptor. BDNF and GDNF are important for growth and survival factors secreted from astrocytes, and BDNF is reported to be reduced in the peripheral blood stream of CM patients. Ang-I/II, angiopoietin-I/II; apoE, apolipoprotein E; AT-1, angiotensin II receptor type 1; BBB, blood brain barrier; BDNF, brain-derived neurotrophic factor; BMEC, brain microvascular endothelial cell; CCL2, C-C Motif Chemokine Ligand 2; CD8^+^, cluster of differentiation 8+; CM, cerebral malaria; CXCL10, chemokines interferon-γ inducible protein 10 kDa; EPCR, endothelial protein C receptor; GDNF, glial cell line-derived neurotrophic factor; gp130, glycoprotein 130 kDa; ICAM-1, intercellular adhesion molecule 1; IFN, interferon; IFNAR, IFN-α receptor; IL, interleukin; IL-6R, interleukin-5 receptor; iRBC, infected red blood cell; LT-α, lymphotoxin-α; LT-βR, lymphotoxin-β receptor; NF-κB, nuclear factor kappa-light-chain-enhancer of activated B cells; PDGFβ, platelet-derived growth factor-β; PDGFRβ, platelet-derived growth factor subunit B receptor; *Pf*EMP-1, *Plasmodium falciparum* erythrocyte membrane protein 1; PTCH1, Patched; SHH, Sonic hedgehog; TGF-β, transforming growth factor-β; TNF, tumor necrosis factor; TNF-α, tumor necrosis factor-α; TNFR-2, TNF receptor 2; VE-cadherin, vascular-endothelial cadherin; VEGF, vascular endothelial cell growth factor.

Key features of the BBB include (1) tight and adherens junction proteins between adjacent endothelial cells, (2) interaction of endothelial cells with the extracellular matrix, a key layer composed of proteins that include collagens and laminins, and (3) modulation of junction proteins by endocytosis and endothelial-expressed matrix metalloproteinases (MMPs) to alter permeability of the membrane as required. However, these properties of the BBB are not intrinsic to BMECs themselves but are controlled by interactions of BMECs with adjacent cells such as pericytes and astrocytes (**Fig 2**).

Pericytes are integral members of the NVU that play a defining role in development and maturation and maintenance of the BBB [5]. Cytoplasmic processes extending from pericytes allow for contact with multiple BMECs [6]. Transcytosis of molecules across the BBB has been shown to be controlled by pericytes, possibly via regulation of BMEC gene expression and polarization of astrocyte endfeet [3]. Indeed, pericytes can promote tight and adherens junction formation via control of BMEC expression of occludin and claudin-5 [7] and vascular-endothelial cadherin (VE-cadherin), respectively. The recruitment of pericytes to the NVU can be mediated by molecules secreted by BMECs, in particular, platelet-derived growth factor-β (PDGFβ), demonstrating an interplay between these two cell types in controlling the properties of the BBB.

Astrocytes are the most abundant glial cells in the brain, with a host of diverse functions that include regulation of the BBB [8]. Specialized endfeet ensheathe BMECs and pericytes and contribute to the maintenance of junction proteins in BMECs. The endfeet are also abundant in the water channel aquaporin-4 and ATP-sensitive potassium channel K^+^ channel subtype 4.1 (Kir4.1) to modulate bulk fluid flow [9]. The powerful influence of astrocytes on the BBB is demonstrated by their ability to induce BBB properties in nonneural endothelial cells [9].

Astrocytes have been shown to be directly involved in host defense [10, 11], as indicated by the functional expression of pattern recognition receptors and immune mediators such as type 1 interferons (IFNs) [12]. Under inflammatory conditions, astrocyte endfeet form their own tight junctions consisting of claudins 1 and 4 to create a separate barrier termed the glial limitans perivascularis [13]. Associated with the parenchymal basal layer of the capillaries and parenchymal blood vessels, the glial limitans functions as a physical barrier to molecules and immune cells crossing the BBB after astrocytes have detected an inflammatory insult. Under conditions of inflammation, both pericytes and astrocytes can induce expression of MMPs on BMECs [7] to induce a leaky barrier that more easily allows incoming leukocytes to traverse the barrier to fight infection. There is extensive molecular and morphological heterogeneity of astrocytes by brain region, with astrocytes at the hindbrain exhibiting stronger antiviral responses than those from other brain regions [12]. Astrocytes with inflammatory (A1) or neuroprotective repair (A2) phenotypes [14] that are akin to M1/M2 macrophages have also been identified. The role of astrocyte heterogeneity in neuroinflammation is still poorly understood.

## How does *Plasmodium*-induced vascular activation lead to breakdown of the BBB?

Permeabilization of the BBB in CM has been demonstrated with increases in albumin, normally partitioned from the brain parenchyma by the BBB, in the cerebral spinal fluid of pediatric patients in Malawi [15]. Current research suggests that vascular activation in the CNS of CM patients begins with iRBCs sequestering to endothelial cells of the BBB, mediated by intercellular adhesion molecule-1 (ICAM-1) and endothelial protein C receptor (EPCR). BMECs recognize sequestered parasites through pattern recognition receptor ligation and signal transduction through ICAM-1 and EPCR. Activation induces phagocytosis of parasites and their products, although BMECs are not normally phagocytic. Astrocyte and microglial cells (glial cells) can also become activated by phagocytosis of parasite-derived vesicles and iRBCs, respectively [16]. Collectively, this results in BMEC-secreted inflammatory cytokines such as TNF and lymphotoxin-α and glial cell secretion of chemokines such as C-X-C motif chemokine ligand 10 kDa (CXCL10) also known as interferon-γ inducible protein-10 (IP-10). Immune cells such as cytotoxic CD8^+^ T cells are attracted to the area. Although research in the *P*. *berghei* ANKA mouse model of CM (ECM) suggests that CD8^+^ T cells are critical in BBB disruption, apoptosis is not a prevalent feature of barrier disruption.

The diffuse barrier breakdown in pediatric CM is rooted in down-regulation of junction proteins, particularly in areas of *P*. *falciparum* sequestration [17]. This results in fluid leakage, vasogenic edema, and herniation of the brain. Noted activation of astrocytes and of glial cells [18] in ECM in mice suggests the involvement of these cells in alteration of BBB permeability [18]. Aquaporin-4, largely expressed on astrocytes, protects against edema in the ECM model [19], demonstrating a role of regulating fluid influx in the brains of *Plasmodium*-infected mice. Models of ECM have also correlated symptoms with reduced levels of astrocyte-produced proteins, such as BMEC-derived brain-derived neurotrophic factor (BDNF) (also observed in pediatric CM patients [20]), suggesting astrocyte dysfunction. Induction of transforming growth factor (TGF-β) in astrocytes in areas of iRBC sequestration has been shown in ECM [21] as well as in CM [22], but little else is known regarding other molecules induced.

## What available models can be used to study the BBB?

Cerebral manifestations of malaria are most often studied using the mouse ECM model, in which vascular permeability and albumin entry into the brain parenchyma has been demonstrated using Evan’s blue. Decoupling the complex interactions between BMEC, pericytes, and astrocytes in vivo is required to understand BBB breakdown in CM. In vivo options for such research include the use of reporter mice to visualize the cells of the BBB by confocal or two-photon microscopy [23, 24]. Debate remains as to the optimal molecules that best characterize the different players involved in the BBB but commonly includes the BMEC markers CD31, Tie 2, and Nestin; astrocyte markers glial fibrillary acidic protein (GFAP) and aldehyde dehydrogenase 1 family member L1 (ALDL1); and pericyte markers platelet-derived growth factor subunit B receptor (PDGFβR) and T-box 18 (TBX-18) [25] (**Fig 1**). Using animals expressing Cre under the promoter for each of these markers, in vivo manipulation of molecules of interest can also be performed, although caution should be employed with regards to knowledge of other cells expressing these molecules in the vasculature of other tissues.

Interactions between the different cell types forming the NVU can also be studied using in vitro culture models. These are by their very nature reductionist, with limited functionality that does not replicate the vast in vivo characteristics of the BBB, particularly the effects of fluid shear stress from blood flow. However, BMECs are generally grown on the surface of permeable supports in a two-compartment transwell system. Addition of other astrocytes and pericytes along with variations of conditioned media can be used to investigate diffusible factors and cell interactions affecting BBB activation and integrity [26]. Aspects of in vitro culture models that are desireable include (1) a high degree of junctional tightness between endothelial cells that is often evaluated by transendothelial electrical resistance (TEER) and permeability dyes with tracer molecules (Lucifer yellow, 444 Da; sodium fluorescein, 377 Da; mannitol, 180 Da), (2) claudin-5 expression, which has been identified as an essential tightening claudin that prevents leakage of small molecules, and (3) appropriate expression, localization, and functionality of efflux transporters from the ABC and SLC family [26]. Engineering culture models with more physiological approximates of the BBB have also been developed, such as the three-dimensional microfluidic chip to incorporate a lumen enclosed by cell-embedded collagen gel. This model can incorporate various cell types, use of fluorescently tagged molecules, and permeability dyes, as well as quantification of cytokine release, but is unable to provide TEER measurements.

## Perspectives

It is now appreciated that diffuse breakdown of the BBB in CM is due to a reduction in the paracellular BMEC junction proteins. However, junction proteins are ultimately controlled by adjacent pericytes and astrocytes; yet there is very little work done characterizing the response of these cell types in malaria. Determination of how astrocytes and pericytes regulate BMEC activation and the expression of junction proteins in the face of *P*. *falciparum* infection is required to develop a more holistic model of CM pathogenesis.
